# The Effect of Zirconia in Hydroxyapatite on *Staphylococcus epidermidis* Growth

**DOI:** 10.1155/2012/432372

**Published:** 2012-08-07

**Authors:** Widowati Siswomihardjo, Siti Sunarintyas, Alva Edy Tontowi

**Affiliations:** ^1^Department of Biomaterials, Faculty of Dentistry, Gadjah Mada University, Yogyakarta 55281, Indonesia; ^2^Department of Biomedical Engineering, School of Graduate Studies, Gadjah Mada University, Yogyakarta 55281, Indonesia; ^3^Department of Mechanical and Industrial Engineering, Faculty of Engineering, Gadjah Mada University, Yogyakarta 55281, Indonesia

## Abstract

Synthetic hydroxyapatite (HA) has been widely used and developed as the material for bone substitute in medical applications. The addition of zirconia is needed to improve the strength of hydroxyapatite as the bone substitute. One of the drawbacks in the use of biomedical materials is the occurrence of biomaterial-centred infections. The recent method of limiting the presence of microorganism on biomaterials is by providing biomaterial-bound metal-containing compositions. In this case, *S. epidermidis* is the most common infectious organism in biomedical-centred infection. *Objective*. This study was designed to evaluate the effect of zirconia concentrations in hydroxyapatite on the growth of *S. epidermidis*. *Methods and Materials*. The subjects of this study were twenty hydroxyapatite discs, divided into four groups in which one was the control and the other three were the treatment groups. Zirconia powder with the concentrations of 20%, 30%, and 40% was added into the three different treatment groups. Scanning electron microscope analysis was performed according to the hydroxyapatite and hydroxyapatite-zirconia specimens. All discs were immersed into *S. epidermidis* culture for 24 hours and later on they were soaked into a medium of PBS. The cultured medium was spread on mannitol salt agar. After incubation for 24 hours at 37°C , the number of colonies was measured with colony counter. Data obtained were analyzed using the ANOVA followed by the pairwise comparison. *Result*. The statistical analysis showed that different concentrations of zirconia powder significantly influenced the number of *S. epidermidis* colony (*P* < 0.05) . *Conclusion*. The addition of zirconia into hydroxyapatite affected the growth of *S. epidermidis*. Hydroxyapatite with 20% zirconia proved to be an effective concentration to inhibit the growth of *S. epidermidis* colony.

## 1. Introduction

Angiogenesis, osteogenesis, and chronic wound healing are natural repairing mechanisms that occur in human body. However, there are some critical defects of size in which these tissues cannot regenerate themselves and need clinical repair [1]. Therefore, the treatment for posttraumatic skeletal conditions such as bone loss is becoming a challenging field to be studied [2]. In most cases, restoration of alignment and stable fixation of the bone is necessary to achieve a successful reconstruction. Bone grafts have an important role in orthopaedic surgery, as well as in the replacement of bone after a trauma or tumour removal [3]. In many cases, adjunctive measures such as bone grafting or bone transports are required to stimulate bone healing and fill the bone defects [2]. Autologous cancellous bone is the most effective biological graft material [4]. It is the most preferable procedure for bone augmentation because of osteoinductive effect and high biocompatibility. However, limitation of available amount and postoperative discomforts including inflammation at donor site are the disadvantages [5]. Skeletal bones comprise mainly of collagen and hydroxyapatite, both are osteoconductive components [1]. Nowadays hydroxyapatite, [Ca_10_(PO_4_)_6_(OH)_2_], as a very important bioceramic is used extensively in medical applications to repair or replace the bone tissues [6, 7]. Dentistry and orthopaedic applications deal a lot with this material because of its good biocompatibility, osteoconductivity, and the bone-bonding properties [8]. Pure hydroxyapatite has chemical composition, biological and crystallographic properties which are highly similar to the bone and the teeth [9].

Hydroxyapatite (HA) powder can be synthesized from various minerals, including coral, gypsum (CaSO_4_.2H_2_O), and calcite [1]. Some methods have been developed in preparing HA powders, the wet methods and solid state reactions. The wet methods can be divided into hydrolysis and hydrothermal techniques. The hydrothermal technique provides HA powder with high degree of crystallinity with Ca-P ratio which is close to the stoichiometric value [10]. It is proved that HA powder can be synthesized from local gypsum using microwave-hydrothermal method [11].

Bulk hydroxyapatite exhibits poor mechanical properties, therefore, for full utilization that must withstand high loads, the improvement of mechanical properties is needed [6]. The primary purpose of adding filler particles is to strengthen a composite [12]. Zirconia is one of the important filler among many fillers or reinforcements, which has been used to increase the strength and toughness of many ceramic materials [7]. In bone surgery zirconia has shown very wide applications [13].

One of the major drawbacks in the use of biomaterials is the occurrence of biomaterial-centred infections [14]. After implantation, the host will interact with biomaterial by forming a conditioning film on its surface. Adherence of microorganisms is mediated by the properties of the biomaterial surface itself. Subsequent surface growth of microorganism will initiate the occurrence of infection. An* in vitro* study proved that the number of microorganism *S. epidermidis* increased in the first 8 to 12 hours after the implantation [14]. Efforts have been done in preventing the contamination of microorganism on the foreign materials during implantation. No doubt that the use of antibiotics might reduce the occurrence of biomaterials-centred infection, but still a significant number of patients are suffering from this condition. The aim of this research was to examine the effect of zirconia on the growth of *S. epidermidis*.

## 2. Materials and Methods

### 2.1. Synthesis of Local Hydroxyapatite and Discs Preparation

The synthesis of hydroxyapatite was conducted and modified [15] to produce powder of hydroxyapatite from local gypsum (Kulon Progo Yogyakarta, Indonesia). The gypsum powder was obtained by pulverizing the gypsum rock. Gypsum powder (20 gr) and 800 mL of 1 M diammonium hydrogen phosphate [(NH_4_)2HPO_4_] were well mixed and treated at 100°C for 20 minutes in a pyrex glass using a microwave digestion system. The system was operated at frequency of 2.45 GHz. After the hydrothermal reaction, reacted sample was washed with distilled water to remove residual ion and dried. The conversion of gypsum to L-HA was estimated from the ratio of X-ray intensities of gypsum peak (*d* = 7.261) and the HA peak (*d* = 2.787) using powder X-ray diffractometry.

Powder of hydroxyapatite (0.4 gr) was put in a mould of compaction instrument [11]. Powder of hydroxyapatite was mixed homogenously with zirconia. In this study the different concentrations of zirconia used were 10%, 20%, 30%, and 40% (weight/weight). Powder of hydroxyapatite-zirconia is put in a mould and pressed with 120 Mpa to produce a disc with a diameter of 10 mm and 3 mm in thickness. Finally, discs were sintered for 2 hours at 1450°C. The sterilization of the hydroxyapatite-zirconia discs was done by keeping the discs in the autoclave for 15 minutes at 121°C. Twenty discs were prepared and divided into five discs for each group of concentration.

### 2.2. Scanning Electron Microscope Examination

The disc specimens were mounted with silver paste on metallic stub. The specimens were then gold coated with a sputtering system under vacuum desiccation. Examination was performed under scanning electron microscope at an acceleration voltage of 7 to 10 KV.

### 2.3. *S. epidermidis* Culturing and Colonies Counting

The* S. epidermidis* (clinical strain) was cultured in mannitol salt agar (MSA) and incubated for 24 hours at 37°C. The cultured bacteria was then transferred into 2 mL of brain heart infusion (BHI) and incubated for 24 hours at 37°C. Solution of NaCl was added into the BHI. Five discs of hydroxyapatite-zirconia from each group of concentration were put into 3 mL culture of* S. epidermidis* in BHI (Brown standard III of 10^8^ CFU/mL). Incubation was followed for 24 hours at 37°C. After incubation, all discs were transferred into 1 mL phosphate buffer saline (PBS) and put on a vibrator to remove the attached *S. epidermidis*. Without taking measurement, all discs were then taken out from the PBS solution, and the solution was diluted up to 10^−4^. Next step, 0.1 mL medium of BHI was transferred into the MSA at the petri dish, using a spreader. The petri dish was incubated for 24 hours at 37°C. The number of colonies was counted using colony counter.

## 3. Discussion

The average and standard deviations of the* S. epidermidis* number of colony on MSA when reacted with hydroxyapatite mixed with zirconia at concentrations of 20%, 30%, and 40% are presented at Table 1.

**Figure 1:**
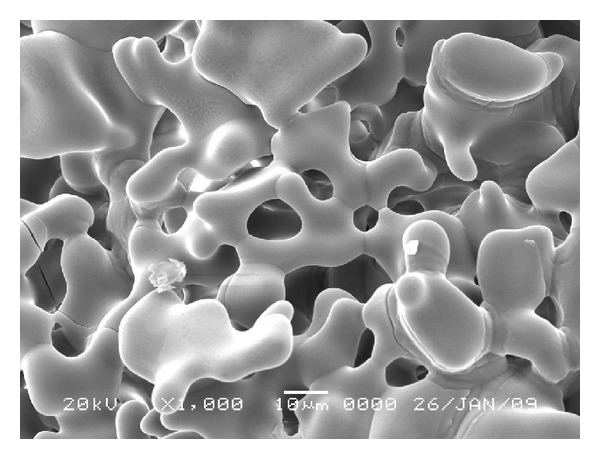
Microstructure of hydroxyapatite.

**Figure 2:**
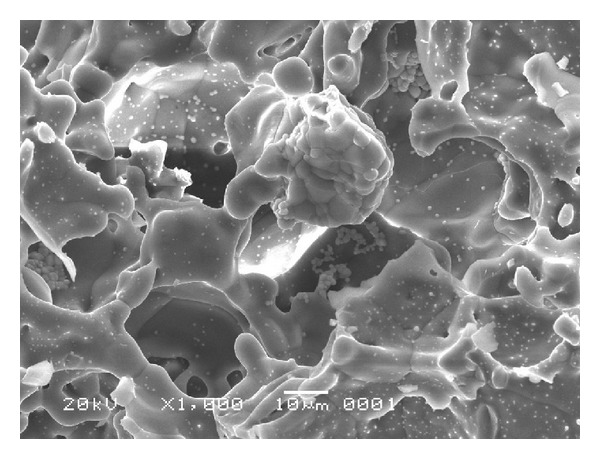
Microstructure of hydroxyapatite with zirconia fill in the pores.

On Table 1 it is noted that the colony number of *S. epidermidis* is consistently influenced by zirconia concentrations in the hydroxyapatite. It is shown that the number of colonies of *S. epidermidis* was smaller as the concentration of zirconia in the hydroxyapatite was increased. In the group of hydroxyapatite without zirconia, it shows the most number of colonies, whereas the concentration of 40% shows the smallest number of colonies. Statistical analysis of this data is carried out using the one-way analysis of variance. This analysis proved whether the different concentrations of zirconia have significant influence on the growth of *S. epidermidis*, and it is indicated by the numbers of the colonies of *S. epidermidis*. The probability from the ANOVA is 0.001, and this value is less then 0.05 confidence levels. From this result it was proved that hydroxyapatite with different concentrations of zirconia had a significant influence on the number of colonies of *S. epidermidis*. The result of this study coincided with the research which stated that the factors influencing bacteria adherence to a biomaterial surface include the surface roughness or physical configuration of the material [16]. The microstructure of hydroxyapatite and hydroxyapatite-zirconia had been tested using scanning electron microscope, and the result proves that hydroxyapatite zirconia has smoother surface roughness than hydroxyapatite [13]. Furthermore, it is stated that the particles of zirconia fill in the pores among the particles of hydroxyapatite [17]. This statement coincides with the scanning electron microscope examination as presented in Figures 1 and 2.

It can be understood that hydroxyapatite-zirconia has less pores compared to hydroxyapatite without zirconia. Material with least pores among particles gives smoother surface of the material. The result of the ANOVA proved that there was significant relation between the zirconia concentrations and the colonies growth of *S. epidermidis* (*P* < 0.05). The number of colonies in hydroxyapatite-zirconia was influenced by the addition of zirconia. This situation related to the statement which mentioned that the present invention provides a method of limiting the presence of a microorganism by contacting the microorganism with material-bound metal-containing compositions [18]. One of the commonly used filler which contain heavy metal is zirconia [12]. The small number of colonies of *S. epidermidis* in hydroxyapatite-zirconia might be assumed due to the toxicity of zirconia as metal. ANOVA analysis was followed by the pairwise comparisons.

The data in Table 2 show significant differences in almost all pair of groups, except the differences between (20–30)% and (30–40)%. Based on this result it can be drawn that zirconia with the concentration of 20% proved to have been an effective concentration in inhibiting the growth of *S. epidermidis.* Moreover, as 20% becomes the effective concentration, a detailed research is still needed. It is important to find out the most effective concentration of zirconia to be added into hydroxyapatite.

## 4. Conclusions

From this study, it can be concluded that concentration of zirconia influenced the number of colonies of *S. epidermidis*. Local hydroxyapatite with 20% zirconia proved to have been an effective concentration in inhibiting the growth of *S. epidermidis*. Scanning electron microscopy examination showed that zirconia filled in the hydroyapatite pores, whereas the less pores the less number of *S. epidermidis* attached.

## Figures and Tables

**Table 1:** Measurement of the number of colony of  *S.* *epidermidis* after being reacted with hydroxyapatite mixed with different concentrations of zirconia.

Replications	Concentrations of zirconia			
	0%	20%	30%	40%
1	30	15	7	3
2	57	16	8	0
3	64	14	10	0
4	32	16	7	0
5	25	12	12	0
*x*	**41.6**	**14.6**	**8.8**	**0.6**
*x* ± *sd*⁡	**41.6 ± 17.61**	**14.6 ± 1.67**	**8.8 ± 2.16**	**0.6 ± 1.34**

**Table 2:** Statistical result of the pairwise comparisons from hydroxyapatite with different concentrations of zirconia on the number of colonies of  *S.* *epidermidis*.

(*I*) VAR0000	(*J*) VAR0000	Mean difference (*I*-*J*)	Std. error	Sig.	95% Confidence interval	
1	1				Lower bound	Upper bound
1.0	2.00	27.00000*	5.65332	.000	15.0155	38.9845
	3.00	32.80000*	5.65332	.000	20.8155	44.7845
	4.00	41.00000*	5.65332	.000	29.0155	2.9845

2.0	1.00	−27.00000*	5.65332	.000	−38.9845	−15.0155
	3.00	5.80000	5.65332	.320	−6.1845	17.7845
	4.00	14.00000*	5.65332	.025	2.0155	25.9845

3.0	1.00	−32.80000*	5.65332	.000	−44.7845	−20.8155
	2.00	−5.8000	5.65332	.320	−17.7845	6.1845
	4.00	8.20000	5.65332	.166	−3.7845	20.1845

4.0	1.00	−41.00000*	5.65332	.000	−52.9845	−29.0155
	2.00	−14.00000*	5.65332	.025	−25.9845	−2.0155
	3.00	−8.20000	5.65332	.166	−20.1845	3.7845

*The mean difference is significant at the 0.05 level.
